# How Difficult Was It? Metacognitive Judgments About Problems and Their Solutions After the Aha Moment

**DOI:** 10.3389/fpsyg.2022.911904

**Published:** 2022-06-22

**Authors:** Nadezhda V. Moroshkina, Alina I. Savina, Artur V. Ammalainen, Valeria A. Gershkovich, Ilia V. Zverev, Olga V. Lvova

**Affiliations:** ^1^The Department of Psychology, St Petersburg University, Saint Petersburg, Russia; ^2^Russian Presidential Academy of National Economy and Public Administration, Moscow, Russia; ^3^HSE Lyceum, National Research University Higher School of Economics, Moscow, Russia

**Keywords:** insight, aha experience, Remote Associates Test, processing fluency, subjective difficulty

## Abstract

The insight phenomenon is thought to comprise two components: cognitive and affective (the Aha! experience). The exact nature of the Aha! experience remains unclear; however, several explanations have been put forward. Based on the processing fluency account, the source of the Aha! experience is a sudden increase in processing fluency, associated with emerging of a solution. We hypothesized that in a situation which the Aha! experience accompanies the solution in, the problem would be judged as less difficult, regardless of the objective difficulty. We also planned to confirm previously discovered associations between the Aha! experience and accuracy, confidence, and pleasure. To test the proposed hypothesis, during the preliminary stage of the study, we developed a set of 100 remote associate problems in Russian (RAT-RUS) and asked 125 participants to solve problems and indicate the Aha! moment (after solution generation or solution presentation), confidence, difficulty, and likability of each problem. As expected, the Aha! experience often accompanied correct solutions and correlated with confidence judgments. We also found a positive correlation between the Aha! experience and problem likability. As for the main hypothesis, we confirmed that the Aha! experience after the presentation of the solution was associated with a decrease in subjective difficulty. When participants could not solve a problem but experienced the Aha! moment after the solution was presented to them, the problem was perceived as easier than one without the Aha! experience. We didn’t find the same effect for the Aha! after solution generation. Thus, our study partially supports the processing fluency account and demonstrates the association between the Aha! experience and metacognitive judgments about the accuracy and difficulty of problems.

## Introduction

Sometimes a solution to a problem suddenly pops into our mind. This moment is called insight. For nearly a century, the phenomenon of insight has attracted the attention of scientists and provoked extensive research. Researchers describe insight as a heterogeneous phenomenon that includes two components: cognitive and affective (Gick and Lockhart, 1995; Danek et al., 2014a). The cognitive component involves a sudden change in the problem representation or the rapid formation of a new concept, which often leads to a solution to the problem (Kounios and Beeman, 2014). The moment of identifying a solution is accompanied by an affective component: the Aha! experience. The Aha! experience is a multidimensional phenomenon that includes a feeling of surprise, suddenness, positive affect, and certainty that the discovered solution is correct (Jung-Beeman et al., 2004; Danek et al., 2014a; Shen et al., 2016). Over the last decade, there has been an increasing interest in the study of the affective component of insight. Some researchers have suggested that the Aha! experience *per se* has independent functions in problem-solving. First of all, the Aha! experience contributes to better memory for the studied material (Auble et al., 1979) or insightful solutions (Danek et al., 2013; Kizilirmak et al., 2016a; Danek and Wiley, 2020). Secondly, the Aha! experience helps to maintain motivation in task performance (Liljedahl, 2005; Oh et al., 2020; Skaar and Reber, 2021). Finally, the Aha! experience can be used as a heuristic in evaluating solution correctness (Tikhomirov and Vinogradov, 1970; Laukkonen et al., 2018) and affects subsequent decision-making processes (Valueva et al., 2016; Laukkonen et al., 2020). Thus, research on the Aha! experience, its possible triggers, and its relationship with metacognitive judgments are relevant to the fundamental theory of thinking and creativity and applied research in pedagogy, marketing, and psychotherapy.

On the surface, data on the Aha! experience research appears counterintuitive. Usually, the more difficult the problem, the lower the confidence in the correctness of the solution (Peterson and Pitz, 1988). However, in the case of insightful problem-solving, the Aha! experience often positively correlates with problem difficulty, as demonstrated by observations of real-life insights (Klein and Jarosz, 2011) and laboratory studies (Webb et al., 2018; Ishikawa et al., 2019). Moreover, when such solutions are found, they seem obvious. Numerous studies have shown that insightful solutions are rated as more confident than are analytical ones (Danek et al., 2014b; Danek and Wiley, 2017; Webb et al., 2018), and these solutions often turn out to be accurate (Salvi et al., 2016; Webb et al., 2016; Danek and Wiley, 2017; Ishikawa et al., 2019). It might be argued that the correlation between the Aha! experience and solution correctness is simply an artifact resulting from the retrospective nature of Aha! experience judgments (e.g., Laukkonen et al., 2020). More precisely, with time to check the solution, participants may reduce their Aha! experience ratings of incorrect solutions. However, results obtained from online measures do not support this argument because they show that the Aha! experience arises before verbalization and verification of the solution (Tikhomirov and Vinogradov, 1970; Laukkonen et al., 2021).

In the following paragraphs, we discuss several hypotheses put forward to explain the nature of the Aha! experience. Most hypotheses are aimed at explaining the correlation between the Aha! experience and the solution accuracy. Danek and Salvi (2020) insist that this correlation lies in the difference between analytical and insightful problem-solving strategies. Consistent with an earlier description (Smith and Kounios, 1996), they suggest that insightful problem-solving is a discontinuous all-or-none mechanism. Because the problem, in this case, is processed unconsciously, the solvers do not have access to any intermediate knowledge or ideas. Therefore, there cannot be an incorrect solution; only correct solutions or no solution at all. In the case of non-insightful solutions, solvers can monitor the solving process by generating incorrect ideas that may be used as suboptimal solutions to avoid timeouts (Smith and Kounios, 1996; Salvi et al., 2016; Danek and Salvi, 2020).

Another explanation that complements the above description supposes that the Aha! experience often accompanies accurate solutions because of their quality (Ohlsson, 1992). The basis of this approach lies in the idea of restructuring, which follows an impasse in the problem-solving process. When the correct idea emerges, the elements of the problem that seemed unrelated suddenly fit together as a whole and form a good “gestalt” because of the restructuring. Accurate solutions bring a sense of closure and pleasure, whereas inaccurate solutions, in which some elements are missing or do not fit, elicit the feeling that the “gestalt” is incomplete (see also Danek and Salvi, 2020).

An alternative theory proposed to explain the phenomenology of insight is the processing fluency account (Topolinski and Reber, 2010), which suggests that the trigger of the Aha! experience is a sudden increase in processing fluency of the entire problem and is associated with the appearance of the solution. Previous studies have shown that the increase in processing fluency induces feelings that are similar to the key features of the Aha! experience; namely, positive affect (Reber et al., 1998; Winkielman and Cacioppo, 2001) and certainty [or the so-called truth effect (Hasher et al., 1977; Brashier and Marsh, 2020)]. In the case of insight, the ease of processing is attributed to the correctness of the solution. In some cases an increase in processing fluency is triggered by irrelevant sources and attributed incorrectly, which leads to false insights (i.e., cases in which the Aha! experience accompanies incorrect solutions; Danek and Wiley, 2017). Unfortunately, Topolinski and Reber (2010) do not explain why, in some cases, the appearance of the solution causes a more abrupt increase in processing fluency than in others and a more intense Aha! experience (see arguments in Laukkonen et al., 2018).

The above literature highlights three possible explanations for the nature of the Aha! experience and its correlation with solution accuracy. The first is based on the idea that there exists a specific method of solution attainment (i.e., a discontinuous all-or-none mechanism). The second account stresses the specific quality of the solution that is achieved as a result of restructuring. The third explanation emphasizes the importance of an abrupt change in the processing fluency of problem features associated with the emergence of a solution. Although the above mentioned theories focus on different aspects of the insightful problem-solving process, they are not mutually exclusive. Additionally, Ohlsson’s (1992) approach predicts that the Aha! experience is caused by the overcoming of the state of impasse, whereas Topolinski and Reber (2010) stress the importance of the shift from low to high processing fluency. Either way, one can expect that the Aha! experience would accompany solutions to difficult problems since the impasse in easy problems is unlikely to appear.

There are just a few studies investigating the relationship between the Aha! experience and the difficulty of problems, and they seem quite contradictory. For example, Webb et al. (2018) reported significant but weak correlations between Aha! experience ratings and solution rates (solvability) of classic insight problems and the English version of the compound remote associate problems. Klein and Jarosz (2011) found a similar relationship when analyzing real-life cases of the Aha! experience. Bilalić et al. (2019) showed that expertise in a given problem contributes to faster solutions while simultaneously diminishing the Aha! experience. In other words, problems become easier for experts, but at the cost of a lower probability of experiencing the Aha! moment. Kizilirmak et al. (2018) also tested the relationship between the objective difficulty of problems and the probability of the occurrence of the Aha! experience. However, they found no effect, neither for successful solution generation (endogenous insights) nor for solution presentation (induced insights). Becker et al. (2020) found an inverse correlation for the German CRAT (Compound Remote Associates Test): an increase in problem difficulty achieved via irrelevant priming led to a decrease in the probability of the Aha! experience emerging. Given this evidence, it is clear that the relationship between the Aha! experience and the difficulty of problems requires further research. In addition to the probability of a solution, the solution time can also indicate the difficulty of a problem. The data on the relation of Aha! experience with response time are controversial. There is evidence that faster responses are more likely to be followed by the Aha! experience (Sandkühler and Bhattacharya, 2008; Cranford and Moss, 2012), as well as evidence that slower responses are more likely to be followed by the Aha! experience (Stuyck et al., 2021).

Gick and Lockhart (1995) suggested that it is not the difficulty of the problem itself that is important but that two conditions must be fulfilled. Initially, the problem should provoke a false representation, which objectively leads to an increase in the solution search time and the feeling of incomprehension; that is, an increase in the problem’s difficulty. Subsequently, when an appropriate representation is found, the solution occurs automatically, which leads to the feeling of suddenness and the perception that the problem is easy. As noted by Gick and Lockhart (1995), many participants, after spending a considerable amount of time searching for the right solution, exclaim in annoyance, when at last they get it, “I’ve been duped!” or “Why didn’t I think of that before?” (p. 199). Moreover, they argue that presenting the correct solution after unsuccessful attempts can provoke a similar Aha! feeling if the solver can easily relate the solution to the problem elements.

Ishikawa et al. (2019) also proposed two conditions that must be met in order for the Aha! experience to occur: first, the problem must have optimal difficulty (Hebb, 1949), and second, the emerged solution must provoke high processing fluency of the entire problem (Topolinski and Reber, 2010). In their experiment, Ishikawa et al. (2019) used confidence ratings as a subjective measure of processing fluency. Results were consistent with the proposed assumption. On the condition that confidence was sufficiently high, the strength of the Aha! experience positively correlated with response times, i.e., problem difficulty. It is important to note that studies of various metacognitive judgments showed that it is not fluency *per se*, but fluency variations can provoke metacognitive experiences (Whittlesea, 2001; Sweklej et al., 2014).

Aside from objective difficulty, there is a subjective feeling of how difficult the problem was to solve. Subjective difficulty is closely associated with the concept of processing fluency and is often described as ease of processing (Topolinski and Reber, 2010). We assume that processing fluency plays an important role in the formation of the Aha! experience. Following previous studies (Gick and Lockhart, 1995; Topolinski and Reber, 2010; Ishikawa et al., 2019), we suppose that two conditions must be met for an Aha! experience to occur: (1) low processing fluency during the early stages of the problem-solving process, which is experienced as a consequence of the initial incompatibility of the problem elements, keeping participants from generating a solution quickly; (2) an increase in processing fluency caused by the attainment of an appropriate representation, which automatically leads to an accurate solution to the problem. Thus, it can be assumed that the Aha! experience is associated not with the difficulty of the problem itself, but with the difference in processing fluency before and after solution generation. If so, the problems that were solved with the Aha! experience would be judged as easier than those with the same objective difficulty but solved without the Aha! experience. Hereafter, we refer to such a situation as the “difficulty estimation bias.”

The proposed explanation can be applied to situations where there is self-generation of a solution (i.e., intrinsic or endogenous insights), as well as to situations in which the correct solution is presented after unsuccessful attempts have been made to solve a problem (i.e., extrinsic or induced insights). Whether these two situations are cognitively and phenomenologically similar is currently being actively discussed (Rothmaler et al., 2017; Kizilirmak et al., 2018; Webb et al., 2019). Our approach suggests that post-solution generation Aha! experiences and post-solution presentation Aha! experiences are partially similar in nature (see also Gick and Lockhart, 1995).

### Research Objectives

The purpose of the study was to investigate the relationship between the Aha! experience (post-solution generation and post-solution presentation) and the difficulty estimation bias (i.e., the tendency to underestimate the difficulty of the problem after the solution is known). We used subjective difficulty judgments after the generation or presentation of the solutions. We also sought to replicate previously reported correlations between the Aha! experience, accuracy, confidence, and pleasure. We tested auxiliary hypothesis to ensure that the created problems induce Aha! experiences similar to those described in previous studies.

The main hypothesis:

The Aha! experience (post-solution generation and post-solution presentation) will decrease the probability of judging the problem as difficult regardless of its objective difficulty level (difficulty estimation bias hypothesis).

The auxiliary hypotheses:

(1) The post-solution generation Aha! experience will positively correlate with judgments about the confidence in the accuracy of the solutions (confidence hypothesis).
(2) The post-solution generation Aha! experience will positively correlate with the objective accuracy of the solutions (accuracy hypothesis).
(3) Problems accompanied by a post-solution generation Aha! experience or a post-solution presentation Aha! experience will be judged more often as pleasant (likability hypothesis).

For the stimuli, we used the Remote Associates Test (RAT; Mednick, 1962; Bowden and Jung-Beeman, 2003). The advantage of remote associate problems is the ability to present problems as a large set during one experimental session (for more details see Bowden et al., 2005). It has also been shown previously that solving problems of this type is usually accompanied by the Aha! experience in about half of the cases, both in situations of solution self-generation and presentation (Kizilirmak et al., 2016b,^2018^).

The existing versions of the Russian-language Remote Associates Test (Voronin and Galkina, 1994; Valueva and Belova, 2011; Toivainen et al., 2019) contain a relatively small number of problems (ranging from 25–48). Therefore, during the preliminary stage of the study, we developed an extended set of problems of the RAT in Russian by applying the principles of problem creation of the previous version of the RAT in Russian (Valueva and Belova, 2011; Valueva and Lapteva, 2021). The main characteristics of the problems are described below (see Supplementary Material 1). We created a database of the extended set of the RAT (RAT-Rus) problems with data on their solvability, induction of Aha! experiences, and other properties to make data available for use by other researchers dealing with Russian-speaking participants (OSF^1^).

## Materials and Methods

### Participants

The study involved 125 volunteers. We did not conduct a power analysis prior to the study. As far as we know, no previous studies have been conducted to identify the effect of our interest (difficulty estimation bias hypothesis), so we do not have relevant data to calculate the sample size. Data from five participants were excluded from the analysis (three participants because of technical reasons and two because of non-adherence to the experimental procedure). Data from 120 participants were used for the analysis (71 female), aged 18–33 years [mean = 24.28; standard deviation (*SD*) = 4.09]. All participants were native Russian speakers. Data were collected both offline before the beginning of the pandemic (78 participants) and online during the pandemic (47 participants). There was no significant difference in average accuracy performance between the two experimental formats: *M*_*online*_ = 0.51 (*SD* = 0.13); *M*_*offline*_ = 0.50 (*SD* = 0.13); *t*(118) = −0.25, *p* = 0.8. For the analysis, we used the combined data obtained online and offline.

### Materials

For the stimuli, we used the RAT-Rus. All problems of the RAT-Rus are triads of cue words that are not directly related to each other. The participants were required to find the target (forth) word to form an expression with each of the words of the triad (idiom, compound term, etc.). For example, for the triad “шоколад, правда, сожалеть” (chocolate, truth, sorry) the answer was “горький” (bitter), with expressions of “горький шоколад” (bitter chocolate), “горькая правда” (bitter truth), “горько сожалеть” (bitterly sorry). A total of 115 triads were composed. Of the total set, 39 problems were taken from the previous RAT version (Valueva and Belova, 2011; Valueva and Lapteva, 2021); the rest were compiled by the authors. Based on the preliminary analysis, 12 problems were excluded, and 100 problems were included in the main set, and three were included in the training set. The triads from the main set were divided into two lists of 50 problems (Lists 1 and 2) so that the words in the triads did not semantically intersect, either with a word in the triad or with a target word. Further details on the principles of creating problems and exclusion criteria are available in Supplementary Material 1.

### Equipment

The offline part of the experiment was carried out using PsychoPy software version 2021.1 (Peirce and MacAskill, 2018), and the online part of the experiment was carried out using PsychoPy version 2021.1 and Pavlovia^2^.

### Procedure

The first part of the data was acquired offline before the pandemic. Participants solved problems independently on a laptop. First, in line with previous research (Jung-Beeman et al., 2004; Kounios et al., 2006; Cranford and Moss, 2012), we presented participants with a detailed description of the Aha! experience. Following the studies by Gick and Lockhart (1995) and Kizilirmak et al. (2016a,2018), we added to the description that the Aha! experience can occur after the correct solution is presented. The resulting description of the Aha! experience was as follows (approximate translation from Russian):

“The Aha! experience is a feeling that you might have when the answer suddenly comes to your mind after making several unsuccessful attempts to solve a problem. You may not be sure how you came up with the answer, but you are relatively confident that it is correct, and you feel positive emotions. The most remarkable illustration of the Aha! experience, as described in the literature, is the case of Archimedes, who suddenly understood how to solve a problem and jumped out of the bath shouting “Eureka!” We do not expect that when solving the following problems, you will experience the same strong feelings; However, if during some of the problems you experience something similar to a sudden insight (e.g., “Aha! Got it!”), mark that you had an Aha! experience. If you do not solve a problem in the allotted time, you will be presented with the correct answer. Please read it and take note of your emotions. If, when you are provided with the correct answer, you experience something similar to the sudden understanding described above (“Oh, exactly! How didn’t I understand it right away?”), please mark it as an Aha! experience.”

After the description of the Aha! experience, participants were presented with instructions on the problems to be solved during the experiment (see Supplementary Material 2). This was followed by a training phase, which consisted of three problems. Then, participants proceeded to the main phase.

**FIGURE 1:**
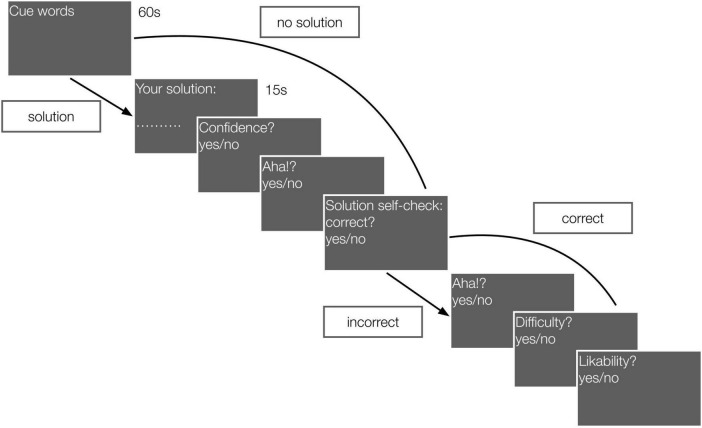
An example of an RAT trial.

In the main phase, each participant solved 50 problems (List 1 or 2). The time flow of one trial is shown in Figure 1. Each triad was presented in the center of the screen in black font on a gray background. The solution time was limited to 1 min. If participants solved the problem before the allotted time, they pressed the SPACE BAR, and a field for entering the discovered solution opened. Then, after entering their solution using the keyboard, the participants were asked to answer closed-ended questions about their confidence in the given solution and whether they had the Aha! experience. Participants used the RIGHT/LEFT arrows to indicate yes/no answers. The correspondence of the LEFT/RIGHT keys to the yes/no responses (for these and all subsequent closed-ended questions) was balanced across participants. If participants could not solve a problem and did not enter any solution, these two questions (confidence and the presence of the Aha! experience) were omitted. The participants were then presented with the correct solution and asked to check whether their own solution matched the correct solution. If they matched, closed-ended questions about whether the problem seemed difficult (yes/no) and whether the participants liked the problem (yes/no) were asked. If participants’ solutions did not match the correct solution or were omitted, participants were asked to note whether they had an Aha! experience when they were presented with the correct solution. If the participant had already found the correct solution on their own, we did not ask about the presence of the Aha! experience a second time (unlike Webb et al., 2019). We were interested in the transition from misunderstanding to understanding. If the person had already understood the solution, we had no reason to expect that the participant would have an Aha! experience after being presented with the solution again. Indeed, the results of the study by Webb et al. (2019) showed that reports of the Aha! experience decline in such scenarios.

After solving all 50 problems, participants received feedback on the number of correct answers. During the experiment, the following behavioral measures were recorded: the accuracy of the answer, the response time, and several subjective judgments: the presence of the Aha! experience (post-solution generation or post-solution presentation), confidence in the given solution, subjective difficulty of the problem, and the likability of the problem.

The experimental program in the online format was the same as that in the offline format. The experiment was carried out with the experimenter observing the participant via video communication. The experimenter communicated with the participants via Skype, Zoom, or Google Meet software and asked them to share their screen. Thus, the experimenter could observe all the actions performed by the participant and conduct a post-experimental interview.

### Data Analysis

#### Data Preprocessing

Participants self-checked the correctness of their solutions by comparing them to the correct solutions. After data collection, experimenters verified participants’ solutions independently, and the results of the verification were included in subsequent data analyses. When participants judged their solutions as incorrect, they were asked about the post-solution presentation Aha! experience. There was a small number of trials in which participants mistakenly assessed their solutions as correct or incorrect. The trials that participants assessed as incorrect but were actually correct were excluded from the analysis of the post-solution presentation Aha! experience (1.2% [75] of all trials). If participants submitted incorrect solutions but assessed them as correct, the post-solution presentation Aha! experience was not measured (2.6% [165] of all trials).

#### Data Analysis Stages

In the first stage of the analysis, the proportion of each type of answer to the problems (correct, intrusion error, or omission error), the mean probabilities, and SDs of all subjective judgments for each type of answer were calculated. We used independent samples t-tests to compare the means.

In the second stage of the analysis, we tested the confidence, accuracy, and likability hypotheses to ensure that the created problems induce Aha! experiences similar to those described in previous studies. We used mixed-effects logistic regression models (with participants and stimuli as random effects). We fitted two logistic mixed-effects regression models, with the post-solution generation and post-solution presentation Aha! experience judgments as outcome variables. In addition, before fitting each model, the predictors were checked for collinearity. To reduce the collinearity of the predictors, we used ridge regressions.

In the third stage of the analysis, we checked the contribution of the Aha! experience and objective difficulty to subjective difficulty judgments to test the difficulty estimation bias hypothesis. We fitted two logistic mixed-effects regression models, with subjective difficulty judgments as the outcome variable: (a) For the first model, only correct answers were selected. The post-solution generation Aha! experience judgments and objective difficulty judgments were included as fixed effects and participants and stimuli were included as random intercepts. (b) For the second model, only incorrect answers were selected. Post-solution presentation Aha! experience judgments and objective difficulty judgments were included as fixed effects and participants and stimuli were included as random intercepts. After building each regression model, a *post hoc* power analysis was conducted for the predictor with the most minor effect.

All data analyses were conducted using the RStudio statistical software (R Studio Team., 2020). The “ppcor” package (Kim, 2015) was used for calculating partial correlations, the “lme4” package (Bates et al., 2015) was used for building the mixed-effect models, and the “simr” package (Green and MacLeod, 2016) was used for conducting power analysis.

## Results

### Descriptive Statistics

The total proportion of correct self-generated solutions was 0.52. The remaining answers were intrusion errors (i.e., incorrect answers; 0.25) and omission errors (i.e., no answer; 0.23). The mean probabilities of each subjective judgment after finding the correct solution, as well as after being presented with the correct solution in unsuccessful generation situations (i.e., intrusion errors and omission errors) are provided in Table 1. Data aggregated for each problem are presented in Supplementary Data 1.

**TABLE 1:** The mean probabilities and standard deviations (SDs) of insight-related affective judgments by answer type (aggregated by stimuli).

Answer type (the proportion of the total number of responses)	The probability of confidence judgments, mean (*SD*)	The probability of post-solution generation Aha! experience, mean (*SD*)	The probability of post-solution presentation Aha! experience, mean (*SD*)	The probability of difficulty judgments, mean (*SD*)	The probability of likeability judgments, mean (*SD*)
Correct (0.52)	0.87 (0.13)	0.56 (0.12)	–	0.23 (0.14)	0.87 (0.07)
Intrusion (0.25)	0.15 (0.16)	0.16 (0.15)	0.63 (0.21)	0.63 (0.20)	0.76 (0.17)
Omission (0.23)	–	–	0.59 (0.26)	0.75 (0.19)	0.70 (0.19)

To compare the probabilities of insight-related affective judgments depending on the type of answer (correct vs. incorrect answers), we (1) combined intrusion and omission errors into a single value of incorrect answers, (2) aggregated the data by triads, and (3) used paired samples *t*-tests. Six triads out of 100 were excluded from comparison of means tests because they had less than 10% correct or incorrect answers.

The mean probability of the post-solution generation Aha! experience did not significantly differ from the mean probability of the post-solution presentation Aha! experience [*t*(93) = −1.3825, *p* = 0.1, Cohen’s *d* = −0.14]. We found that unsolved and incorrectly solved problems were judged as difficult more often than were correctly solved problems [*t*(93) = −28.881, *p* < 0.001, Cohen’s *d* = −2.98]. We also found that correctly solved problems were judged more often than unsolved or incorrectly solved problems as likable [*t*(93) = 8.5735, *p* < 0.001, Cohen’s *d* = 0.88].

### Relationship Between the Post-solution Generation Aha!-Experience, Other Subjective Judgments, and Accuracy

For the analysis of the relationship between the post-solution generation Aha! experience judgment, other measured subjective judgments, and the accuracy of the solution, we selected only those cases in which the participant was asked about the presence of the post-solution generation Aha! experience (i.e., the participant provided an answer [correct or incorrect]). The logistic mixed-effects regression model was fitted to the post-solution generation Aha! experience judgment as a binary dependent variable. The model included the following predictors: correctness (correct/incorrect answers), confidence, subjective difficulty, response time, likability of the problem, and the intercept for participants and stimuli. The model also included the interaction of response time and correctness. The collinearity analysis before building the model revealed significant evidence of collinearity of predictors (see Table 2 for predictor correlations). To reduce collinearity, the confidence predictor was regressed against correctness, subjective difficulty was regressed against confidence, and response time was regressed against correctness. The model showed positive effects of four predictors: correctness, confidence, subjective difficulty and likability of the problem (Table 3). The relationship between the probability of post-solution generation Aha! experience and response time was not found. However, correct solutions that took longer to be found were more often accompanied by the Aha! experience. For incorrect solutions, we observed an inverse relationship, as evidenced by the significant interaction between the response time and correctness predictors.

**TABLE 2:** Predictor correlations for the post-solution generation Aha! experience model.

	Correctness	Confidence	Subjective difficulty	Response time
Confidence	0.772***			
Subjective difficulty	−0.491***	−0.516***		
Response time	−0.503***	−0.457***	0.292	
Likability	0.119	0.149	–0.102	−0.072

***p < 0.001.

**TABLE 3:** Post-solution generation Aha! experience analysis (Correctness + Subjective difficulty + Confidence + Response time + Likability).

Predictor	Coef. β	*SE*	*z*	*p*
Intercept	−3.65	0.23	−16.19	<0.001***
Correct solution	18.98	0.14	21.38	<0.001***
Confidence	12.31	0.16	16.13	<0.001***
Subjective difficulty	1.32	0.13	2.18	0.03*
Likability	3.82	0.15	9.17	<0.001***
Response time	−0.99	0.01	−1.49	0.14
Correct solution*Response time	1.06	0.01	7.56	<0.001***
Model parameters	Pseudo-*R*^2^	AIC	BIC	logLik
	0.62	3792.2	3848.8	−1887.1

Power for Subjective difficulty predictor: 100% (69.15, 100.00). *p < 0.05; ***p < 0.001.

Thus, the accuracy, confidence, and likability hypotheses were supported. There was also evidence of a weak relationship between the Aha! experience and the subjective difficulty of problems, even though difficulty was negatively correlated with confidence.

### The Relationship Between the Post-solution Presentation Aha! Experience, Subjective Difficulty, Likability, and Error Type

To model the relationship between the post-solution presentation Aha! experience and other measurements, we selected only the trials with omission and intrusion errors. We built a logistic mixed-effects regression model, with Aha! experience judgments as a binary dependent variable. The model included the following predictors: error type, subjective difficulty, likability, and intercept for participants and stimuli. A collinear analysis was performed, which did not reveal significant evidence for the collinearity of predictors (*r* = 0.169). The final model was built using a backward selection method and showed a negative effect of subjective difficulty and a positive effect of likability of the problems (Table 4). Results showed that post-solution presentation Aha! experiences occurred in situations where participants rarely judged the problem as subjectively difficult and more often judged the problem as likable.

**TABLE 4:** Post-solution presentation Aha! experience analysis (Subjective difficulty + Likability).

Predictor	Coef. β	*SE*	*z*	*p*
Intercept	−0.92	0.18	−5.03	<0.001***
Subjective difficulty	−0.81	0.13	−6.26	<0.001***
Likability	16.81	0.14	20.38	<0.001***
Model parameters	Pseudo-*R*^2^	AIC	BIC	logLik
	0.49	2732	2761.5	−1361

Power for Subjective difficulty predictor: 100% (83.16, 100.00). ***p < 0.001.

### Contributions of the Aha! Experience and Objective Difficulty of the Problems to Subjective Difficulty Judgments

To clarify the relationship between subjective difficulty judgments and the Aha! experience, we added objective difficulty as a predictor of subjective difficulty judgments to control its effect. We expected that the Aha! experience would induce a decrease in the probability of subjective difficulty judgments, regardless of the objective difficulty of the problems.

To perform this analysis, we built two logistic mixed-effects regression models for correct and incorrect answers, with subjective difficulty as a binary dependent variable. The first model for correct answers included post-solution generation Aha! experience judgments and objective difficulty as predictors. The second model for incorrect answers included post-solution presentation Aha! experience judgments and objective difficulty as predictors. Random intercepts for participants and stimuli were added. In addition to these complete models (Tables 5, 6), we built depleted models with only one predictor and compared it with depleted models without an interaction to evaluate the contribution of the main effects of Aha! experiences and objective difficulty. To evaluate the interaction effect, we compared the complete models with models without an interaction.

**TABLE 5:** Subjective difficulty analysis (Post-solution generation Aha! experience + Objective difficulty).

Predictor	Coef. β	*SE*	*z*	*p*
Intercept	−2.77	0.24	−11.51	<0.001***
Objective difficulty	2.40	0.44	5.40	<0.001***
Post-solution generation Aha!	0.30	0.23	1.33	0.18
Objective difficulty*Post-solution generation Aha!	−2.10	0.48	0.44	0.66
Model parameters	Pseudo-*R*^2^	AIC	BIC	logLik
	0.34	2723.8	2753.9	−1356.9

Power for Post-solution generation Aha! predictor: 24.5% (18.71, 31.06). ***p < 0.001.

**TABLE 6:** Subjective difficulty analysis (Post-solution presentation Aha! experience + Objective difficulty).

Predictor	Coef. β	*SE*	*z*	*p*
Intercept	0.97	0.31	3.15	0.002**
Objective difficulty	1.69	0.46	3.64	<0.001***
Post-solution presentation Aha!	−0.77	0.33	−2.32	0.02*
Objective difficulty*Post-solution presentation Aha!	−0.54	0.53	−1.02	0.31
Model parameters	Pseudo-*R*^2^	AIC	BIC	logLik
	0.39	2733.2	2768.2	−1360.6

Power for Post-solution presentation Aha! predictor: 64% (56.93, 70.56). *p < 0.05; **p < 0.01; ***p < 0.001.

In the first model, the main effect of the post-solution generation Aha! experience was not significant [χ^2^(1) = 3.47, *p* = 0.06]; however, the main effect of objective difficulty was significant [χ^2^(1) = 35.31, *p* < 0.001]. The interaction between objective difficulty and the post-solution generation Aha! experience was not significant [χ^2^(1) = 1.18, *p* = 0.66]. Thus, a significant correlation between the post-solution generation Aha! experience and probability of subjective difficulty judgments for correctly solved problems was not found. Objective difficulty was the only significant predictor of subjective difficulty judgments. The *post hoc* power analysis for the post-solution generation Aha! predictor showed 24.5% (Table 5).

In the second model, the main effects of both the post-solution presentation Aha! experience and objective difficulty were significant [χ^2^(1) = 88.20, *p* < 0.001 and χ^2^(1) = 16.66, *p* < 0.001, respectively]. The interaction between objective difficulty and the post-solution presentation Aha! experience was not significant [χ^2^(1) = 1.01, *p* = 0.31]. There was a significant correlation between the post-solution presentation Aha! experience and probability of subjective difficulty judgments for incorrectly solved problems. The probability of judging a problem as subjectively difficult was reduced in situations accompanied by a post-solution presentation Aha! experience. The *post hoc* power analysis for the post-solution presentation Aha! predictor showed 64% (Table 6).

## Discussion

The purpose of the study was to investigate how the Aha! experience relates to accuracy, confidence, likability, and the objective and subjective difficulty of the problems. According to our approach, subjective difficulty judgments reflect processing fluency, which is the source of the Aha! experience. This is why the relationship between the Aha! experience and subjective difficulty judgments was of specific interest.

### The Probability of Inducing Aha Experience in the Remote Associates Test in Russian

To test our hypotheses, we developed a set of remote associate problems in Russian. Results showed that the average percentages of correctly solved problems with the Aha! experience and those without the Aha! experience in the current study closely align with those reported in previous studies. These results are similar to those obtained using the English CRAT (Kounios et al., 2006; Cranford and Moss, 2012) and German CRAT (Kizilirmak et al., 2016b), which showed that the Aha! experience accompanies about half of all correct solutions. Therefore, our Russian version of the RAT seemed to similarly trigger each solution type (insightful/non-insightful) as was demonstrated with previously validated CRATs. Furthermore, in the study by Kizilirmak et al. (2016b), it was found that the post-solution presentation Aha! experience occurs even more often than the post-solution generation Aha! experience, although an opposite trend was obtained in their subsequent study (Kizilirmak et al., 2018). The study by Webb et al. (2019) using the English CRAT found no significant differences between the ratings of the post-solution generation Aha! experience and the post-solution presentation Aha! experience. Similarly, we did not find significant differences between the likelihood of having a post-solution generation Aha! experience and having a post-solution presentation Aha! experience. However, the procedures in our study differed from those of the studies mentioned above: we did not ask participants whether the presented solution was plausible. Therefore, we did not exclude from the analysis cases in which participants did not understand the presented solutions.

### The Correlation of the Aha! Experience With the Accuracy, Confidence, Likability of Problems and Response Time

Based on previous research, we hypothesized that the Aha! experience is associated with the accuracy, confidence, and likability of problems. Analysis of self-generated solutions showed that the Aha! experience occurs more often when participants found a correct solution than when they found an incorrect solution. This result is in line with previous studies demonstrating a positive relationship between the Aha! experience and accuracy on the CRAT and other types of problems (Salvi et al., 2016; Webb et al., 2016; Danek and Wiley, 2017; Ishikawa et al., 2019) but not with the results of Stuyck et al. (2021) who used the Dutch CRAT. Additionally, we revealed an interaction effect of response type (correct/incorrect) and response time for the appearance of the Aha! experience. There was a positive correlation between the Aha! experience and response time for correct solutions; however, this correlation was negative for incorrect solutions. This may be due to the following reasons. Firstly, some participants tended to provide incorrect answers immediately before the trial timed out to avoid not making a response (for more details, see Salvi et al., 2016). Kounios et al. (2008) associated such incorrect answers with analytical strategy. In such cases, participants assume that the answer would be incorrect (e.g., their answer connects with only one cue word) and do not report the Aha! experience because they sense an incongruity in the representation of the problem. Thus, the Aha! experience rarely accompanies incorrect answers that are entered close to the deadline. Secondly, the Aha! experience arises more often when participants identify an answer after having spent a considerable amount of time searching for the correct solution. This can be explained by the fact that quick correct solutions are more commonly found for simple problems. In such situations, there is no increase in processing fluency because there is no feeling of incomprehension when dealing with the problem.

It is worth noting reports by several studies that faster responses are more often followed by the Aha! feeling (e.g., Sandkühler and Bhattacharya, 2008; Cranford and Moss, 2012), which is inconsistent with our data. However, there are also other studies that have not reported such a tendency (e.g., Jung-Beeman et al., 2004; Hedne et al., 2016; Stuyck et al., 2021). Cranford and Moss (2012) distinguished between immediate insights, when the first guess is correct, and non-immediate insights, when the first guess is incorrect and the solution is found via restructuring. Because our description of the Aha! experience stated that insight can occur after several unsuccessful solution attempts, participants were more likely to report non-immediate insights.

Our data support the hypothesis that the Aha! experience correlates with confidence judgments, and this result is consistent with previous studies (Hedne et al., 2016; Danek and Wiley, 2017). In addition, we found that the Aha! experience positively correlated with response time and problem difficulty. At the same time, confidence judgments negatively correlated with problem difficulty. Thus, confidence and Aha! experience judgments at least partially rely on different sources.

We found a positive correlation between the probability of having an Aha! experience and problem likability for both self-generated and presented solutions. This is consistent with previous studies showing a close relationship between the Aha! experience and positive emotions (Danek et al., 2014a; Ishikawa et al., 2019), although we asked participants about the likability of the problems and not about the pleasure of finding a solution. Nevertheless, we noted a relatively high level of likability for all our problems. Moreover, the problems were liked more often when the correct solution was generated (0.87) than when they were presented (0.76 after intrusion and 0.66 after omission). This discrepancy may be attributed to participants’ incomprehension of several of the presented solutions. Furthermore, participants may have been dissatisfied when they were unable to solve the problem on their own, which reduced their likability judgments.

### The Relations of the Aha! Experience and the Difficulty Estimation Bias

Considering that our results are largely consistent with previous studies, our main hypothesis (i.e., if the Aha! experience is associated with an increase in processing fluency, then problems solved with the Aha! experience should seem subjectively less difficult when the answer is known, be it generated or presented) can be discussed. To test this hypothesis, we compared the probability that the problem would be judged as difficult after Aha! and no-Aha! experience solutions. It is important to note that we controlled the objective difficulty of the problems. Successful (i.e., an Aha! experience after generation) and unsuccessful (i.e., an Aha! experience after presentation) solutions were analyzed separately. We did not find a correlation between the post-solution generation Aha! experience and subjective difficulty judgments, which may have been due to the overall low rate of “difficult” judgments for successfully solved problems (average probability = 0.23). Our results revealed a correlation between the post-solution presentation Aha! experience and subjective difficulty judgments. Problems with the same objective difficulty (solution rate) were judged as difficult less often if the presentation of the solutions induced an Aha! experience than when they induced no Aha! experience. Thus, our results support the notion that the post-solution presentation Aha! experience is associated with the difficulty estimation bias; problems seem easier because the presentation of solutions increases processing fluency. We should note that the *post hoc* power analysis results indicate 64% for the post-solution presentation Aha! which can be considered as medium power. and 24.5% for the post-solution generation Aha! demonstrating that the sample size could be insufficient to make final conclusions. In addition to possible undersampling, we should mention some details in our experimental procedure. Significantly more time passed between the post-solution generation Aha! judgment and the difficulty judgment then it passed between the post-solution presentation Aha! judgment and the difficulty judgment. Because of this difference, the effect of the fluency variation may not have shown up. Apart from procedural reasons, lack of the effect may also indicate that these two experiences are different in their nature (see also Rothmaler et al., 2017). Further research is needed to test these assumptions.

Our results indicate a similarity between the post-solution presentation Aha! experience and the I-knew-it-all-along phenomenon (also known as hindsight bias), which refers to the tendency for people to falsely believe that they could predict the outcome of an event once it has become known (Fischhoff, 1977; Hasher et al., 1981). It must be emphasized that both phenomena reflect an illusory feeling of simplicity, even though the objective difficulty of a problem is high. Where does such illusory simplicity of the problem originate? The processing fluency account only postulates that there is a shift in processing fluency when obtaining a solution; however, it does not explain its cognitive mechanisms. We suggest, with reference to the Problem Space Theory (Newell and Simon, 1972), that the difficulty estimation bias occurs if the path from the goal state to the initial state is more accessible than the path from the initial state toward the goal state. In remote associate problems specifically, the associative strength between the cue words and the target may be weaker than that between the target and the triad of cue words. Therefore, when the search is initiated from the cue words, unsuitable associations are activated first, whereas when the search is initiated from the target word, the connection with the triad of cue words is obvious. This interpretation can be verified using linguistic corpus data.

The subjective difficulty of problems is rarely included as a measurable variable in insight research. However, our results showed that it is an informative indicator. Previously, researchers have focused on examining the relationship between the Aha! experience and the objective difficulty of problems (i.e., solution probability and solution time; Kizilirmak et al., 2018; Webb et al., 2018; Ishikawa et al., 2019; Becker et al., 2020). Although subjective difficulty is associated with objective difficulty, it is not entirely derived from objective difficulty. Moreover, our study demonstrated that the difference between these two measures is an important characteristic of insight phenomenology. In addition, according to our approach, the difference between pre- and post-solution subjective difficulty judgments should be higher for problems solved with the Aha! experience than for those solved without the Aha! experience (because of the drastic shift in processing fluency). In a recent study by Stuyck et al. (2021), participants rated the subjective difficulty (from 0 to 100) of CRAT problems 2 s after the start of the trial and after the solution was obtained. They found a positive correlation between the Aha! experience and the initial difficulty judgments but not the final judgments. We analyzed their data to test whether the Aha! experience had an influence on the magnitude of the decline in subjective difficulty ratings at the end of the trial compared with that at the start by performing mixed-effects linear regression with the difference between the second and first ratings as the outcome variable, the Aha! experience as a fixed effect, and participants and stimuli as random effects. The results corroborated our hypothesis: there was a significant effect of the Aha! experience judgment (mean difference between the second and first difficulty ratings: *M*_*aha*_ = −25.4, *M*_*no aha*_ = −12.8; *B* = 12.85, standard error = 1.8, *t* = 7.141, *p* < 0.001). This suggests that the decline in difficulty ratings was more marked for problems solved with the Aha! experience than for those solved without the Aha! experience. Thus, our study and that of Stuyck et al. (2021) showed that the change in processing fluency associated with insightful solutions can be attributed to the subjective difficulty of the problem.

## Limitations

The use of subjective reports in the study imposes particular constraints on the interpretation of the results. The procedure of providing participants with the definition of the Aha! experience is common for insight studies because, without it, participants might not understand what exactly they have to report. Nevertheless, the definition includes different dimensions, such as suddenness, impasse, pleasure, and certainty, that might have prompted participants to use them when evaluating whether or not the solution was insightful. It raises the question whether it is reasonable to use certainty and pleasure as predictors of the global “Aha!” if these dimensions are included in the very definition of this experience. We are aware of this problem, however, we purposely used the same paradigm as Kizilirmak et al., 2016b and Webb et al. (2019) as we wanted to know whether our problems induce Aha! experiences similar to those described in previous studies. Similarly, the issue of using different scales requires a closer look. Following the tradition of Bowden et al. (2005), we used binary scales for Aha! experience, as well as for the other subjective measures. However, these experiences, especially certainty and subjective difficulty, obviously lay within some sort of continuum. It is the question of further research, whether the same effects would be obtained if the continuous scales were used. Another limitation comes from the fact that participants in our study reported subjective difficulty only once at the end of the trial. To further clarify the relationship between Aha! and difficulty estimation bias, studies can be conducted with subjective difficulty judgments at different problem-solving stages.

## Conclusion

We aimed to examine how Aha! experience relates to the subjective difficulty judgments about the problems. Based on the approach of Gick and Lockhart (1995) and the processing fluency account (Topolinski and Reber, 2010), we assumed that the Aha! experience arises from an abrupt change in the processing fluency of a problem at the time of solution appearance (generation or presentation). Accordingly, we hypothesized that Aha! experience would lead to underestimating the difficulty of the problems, i.e., the difficulty estimation bias. The results showed that post-solution presentation Aha! experience was associated with a decrease in the subjective difficulty of the problem while post-solution generation Aha! experience was not. In other words, although the solver could not find the solution to the problem on their own if the Aha! experience accompanied the presentation of the solution, the problem was perceived as easy and that they must have solved it. We consider this an illusory feeling, which makes the post-solution presentation Aha! experience similar to the I-knew-it-all-along phenomenon (hindsight bias). However, further research is required to test the similarity of these phenomena. To the best of our knowledge, only a few studies (Hoch and Loewenstein, 1989; Ash and Wiley, 2008) have demonstrated the emergence of hindsight bias in insight problem-solving.

In summary, we highlight the importance of studying the relationship between the phenomenology of insight and the subjective difficulty of problems. We urge future research to include not only self-report measures of the discovered solution but also those of the problems being solved. The processing fluency account suggests that the feeling of fluency is attributed to different sources; however, this idea remains poorly studied on the problems that induce insight. We also emphasize the importance of investigating the connection between the Aha! experience and other metacognitive experiences and phenomena and the inclusion of the Aha! experience in a broader context of theories on metacognitive regulation.

## Data Availability Statement

The datasets presented in this study can be found in online repositories. The names of the repository/repositories and accession number(s) can be found in the article/Supplementary Material.

## Ethics Statement

The studies involving human participants were reviewed and approved by Office for Human Research Protections (OHRP). The patients/participants provided their written informed consent to participate in this study.

## Author Contributions

NM: supervision, conceptualization, development of stimulus material, methodology, and writing – original draft. AS: conceptualization, data collection, formal analysis, visualization, and writing – original draft. AA: conceptualization, methodology, data collection, formal analysis, and writing – review and editing. VG: conceptualization, development of stimulus material, methodology, and writing – review and editing. IZ: conceptualization, data collection, formal analysis, and writing – review and editing. OL: conceptualization, data collection, and writing – review and editing. All authors contributed to the article and approved the submitted version.

## Conflict of Interest

The authors declare that the research was conducted in the absence of any commercial or financial relationships that could be construed as a potential conflict of interest.

## Publisher’s Note

All claims expressed in this article are solely those of the authors and do not necessarily represent those of their affiliated organizations, or those of the publisher, the editors and the reviewers. Any product that may be evaluated in this article, or claim that may be made by its manufacturer, is not guaranteed or endorsed by the publisher.

## Footnotes

1
https://osf.io/zumnr/

2
http://pavlovia.org/
