# Supplementary material for: How Difficult Was It? Metacognitive Judgments About Problems and Their Solutions After the Aha Moment
Source: Front Psychol. 2022 Jun 22;13:911904. doi: 10.3389/fpsyg.2022.911904 (PMC9258945; doi:10.3389/fpsyg.2022.911904)
Supplement: Supplementary file 1 [file Table_1.DOCX]

***Supplementary Material***

# Supplementary Data 1

|  |  | | Measured out of all attempts | | | | | | Measured out of correctly solved | | | Measured out of unsolved or incorrectly solved |
| --- | --- | --- | --- | --- | --- | --- | --- | --- | --- | --- | --- | --- |
| # | Problem | Correct Solution | Accuracy | Probability of omissions | Probability of intrusions | Probability of difficulty judgments | Probability of likability judgments | Number of participants attempted the problem | Median RT (sec) | Proportion of Aha reports | Proportion of confidence reports | Proportion of post-presentation Aha! reports |
| 1 | терпение голос внешность | ангельский | 0.28 | 0.27 | 0.45 | 0.60 | 0.78 | 60 | 7.23 | 0.65 | 0.94 | 0.66 |
| 2 | отдых данные военный | база | 0.12 | 0.37 | 0.51 | 0.58 | 0.78 | 65 | 8.97 | 0.5 | 1 | 0.71 |
| 3 | дробь перепонка установка | барабанная | 0.82 | 0.08 | 0.1 | 0.15 | 0.88 | 60 | 6.76 | 0.55 | 1 | 0.91 |
| 4 | танец стих медведь | белый | 0.59 | 0.16 | 0.26 | 0.26 | 0.86 | 58 | 8.52 | 0.47 | 0.91 | 0.73 |
| 5 | синий отпустить анекдот | борода | 0.52 | 0.2 | 0.28 | 0.57 | 0.85 | 60 | 8.86 | 0.71 | 0.97 | 0.69 |
| 6 | расчет заводской гражданский | брак | 0.55 | 0.08 | 0.37 | 0.35 | 0.87 | 60 | 8.65 | 0.58 | 0.97 | 0.88 |
| 7 | стакан магнитный солнце | буря | 0.32 | 0.38 | 0.3 | 0.67 | 0.68 | 60 | 9.5 | 0.47 | 0.74 | 0.52 |
| 8 | гусь хлеб живой | вода | 0.29 | 0.39 | 0.32 | 0.61 | 0.59 | 59 | 11.54 | 0.53 | 0.71 | 0.38 |
| 9 | деньги потерянный приходить | время | 0.42 | 0.28 | 0.30 | 0.63 | 0.68 | 60 | 11.73 | 0.44 | 0.68 | 0.62 |
| 10 | забить программа железный | гвоздь | 0.85 | 0.1 | 0.05 | 0.13 | 0.88 | 60 | 7.42 | 0.55 | 0.98 | 0.88 |
| 11 | лоб алмаз стрелять | глаз | 0.46 | 0.18 | 0.36 | 0.64 | 0.69 | 61 | 17.55 | 0.5 | 0.68 | 0.38 |
| 12 | сложить светлый снег | голова | 0.08 | 0.53 | 0.38 | 0.82 | 0.5 | 60 | 20.39 | NA | NA | 0.23 |
| 13 | кровь мечта река | голубая | 0.45 | 0.15 | 0.4 | 0.48 | 0.83 | 60 | 16.09 | 0.56 | 0.85 | 0.61 |
| 14 | шоколад правда сожалеть | горький | 0.93 | 0.02 | 0.05 | 0.08 | 0.87 | 60 | 6.14 | 0.46 | 1 | NA |
| 15 | петух морской волосы | гребешок | 0.47 | 0.19 | 0.34 | 0.53 | 0.81 | 59 | 08.02 | 0.71 | 0.96 | 0.73 |
| 16 | молоко клетка ребенок | грудной | 0.3 | 0.42 | 0.28 | 0.48 | 0.92 | 60 | 14.34 | 0.56 | 0.94 | 0.81 |
| 17 | князь валяться лечебный | грязь | 0.53 | 0.23 | 0.23 | 0.45 | 0.8 | 60 | 5.55 | 0.47 | 1 | 0.73 |
| 18 | надуть алый бантик | губы | 0.44 | 0.19 | 0.37 | 0.39 | 0.8 | 59 | 08.05 | 0.69 | 1 | 0.78 |
| 19 | шляпа табак техника | дело | 0.25 | 0.47 | 0.28 | 0.78 | 0.57 | 60 | 19.88 | 0.67 | 0.93 | 0.23 |
| 20 | косой грибной четверг | дождь | 0.92 | 0.05 | 0.03 | 0.2 | 0.85 | 60 | 5.11 | 0.4 | 0.91 | NA |
| 21 | животное задание арест | домашний | 0.41 | 0.28 | 0.3 | 0.37 | 0.83 | 46 | 12.75 | 0.58 | 0.95 | 0.81 |
| 22 | скатерть присесть проложить | дорога | 0.93 | 0.03 | 0.03 | 0.18 | 0.9 | 60 | 7.64 | 0.61 | 0.91 | NA |
| 23 | барабанный оружейный математический | дробь | 0.58 | 0.12 | 0.3 | 0.37 | 0.92 | 60 | 07.09 | 0.6 | 0.91 | 0.74 |
| 24 | задать птица пыл | жар | 0.8 | 0.13 | 0.07 | 0.22 | 0.9 | 60 | 6.98 | 0.67 | 0.98 | 0.82 |
| 25 | дорога занавес логика | железный | 0.38 | 0.25 | 0.37 | 0.52 | 0.95 | 60 | 8.87 | 0.7 | 0.91 | 0.89 |
| 26 | семь рот висячий | замок | 0.26 | 0.56 | 0.18 | 0.74 | 0.72 | 50 | 12.12 | 0.46 | 0.77 | 0.33 |
| 27 | искушать воздушный зеленый | змей | 0.97 | 0.03 | 0 | 0.13 | 0.92 | 39 | 6.4 | 0.5 | 0.92 | NA |
| 28 | молодежь руки век | золотой | 0.67 | 0.23 | 0.1 | 0.27 | 0.87 | 60 | 6.96 | 0.68 | 1 | 0.79 |
| 29 | мудрость точить заговаривать | зуб | 0.83 | 0.03 | 0.13 | 0.15 | 0.9 | 60 | 6.3 | 0.64 | 1 | 0.8 |
| 30 | коса почка заложить | камень | 0.38 | 0.47 | 0.15 | 0.6 | 0.7 | 60 | 7.71 | 0.57 | 0.78 | 0.32 |
| 31 | хранение велосипедный скрытый | камера | 0.27 | 0.27 | 0.46 | 0.53 | 0.81 | 59 | 08.04 | 0.62 | 1 | 0.82 |
| 32 | последний роса нос | капля | 0.32 | 0.5 | 0.18 | 0.65 | 0.75 | 60 | 13.85 | 0.47 | 0.89 | 0.61 |
| 33 | капуста мина почва | кислый | 0.28 | 0.25 | 0.47 | 0.53 | 0.72 | 60 | 12.33 | 0.59 | 0.88 | 0.68 |
| 34 | грудной живой тетрадь | клетка | 0.47 | 0.27 | 0.27 | 0.63 | 0.8 | 60 | 14.39 | 0.68 | 0.79 | 0.68 |
| 35 | вода момент игрок | ключевой | 0.12 | 0.35 | 0.53 | 0.68 | 0.82 | 60 | 12.35 | 0.57 | 1 | 0.68 |
| 36 | белка обозрение пятый | колесо | 0.7 | 0.13 | 0.17 | 0.3 | 0.82 | 60 | 07.06 | 0.67 | 0.95 | 0.65 |
| 37 | газетный газовый музыкальный | колонка | 0.1 | 0.25 | 0.65 | 0.65 | 0.75 | 60 | 31.44 | 1 | 1 | 0.54 |
| 38 | сводить венец свет | конец | 0.78 | 0.12 | 0.1 | 0.44 | 0.73 | 59 | 9 | 0.37 | 0.59 | 0.5 |
| 39 | зуб квадратный рубить | корень | 0.7 | 0.13 | 0.17 | 0.37 | 0.93 | 60 | 6.94 | 0.6 | 0.9 | 0.71 |
| 40 | слово проблема зло | корень | 0.33 | 0.31 | 0.36 | 0.59 | 0.79 | 39 | 7.6 | 0.46 | 0.77 | 0.65 |
| 41 | седло слизнуть лёд | корова | 0.12 | 0.57 | 0.31 | 0.8 | 0.51 | 65 | 11.33 | 0.75 | 0.75 | 0.22 |
| 42 | лев голый ужин | король | 0.62 | 0.3 | 0.08 | 0.55 | 0.77 | 60 | 13.24 | 0.51 | 0.68 | 0.35 |
| 43 | кожа слоновый лечь | кость | 0.52 | 0.27 | 0.22 | 0.65 | 0.77 | 60 | 13.32 | 0.52 | 0.65 | 0.41 |
| 44 | ученый мешок хвост | кот | 0.85 | 0.07 | 0.08 | 0.1 | 0.83 | 60 | 4.19 | 0.47 | 0.94 | 0.38 |
| 45 | ад ходить друг | круг | 0.72 | 0.17 | 0.12 | 0.43 | 0.8 | 60 | 8.65 | 0.37 | 0.58 | 0.33 |
| 46 | валюта корабль учебный | курс | 0.46 | 0.29 | 0.25 | 0.44 | 0.85 | 59 | 8.14 | 0.56 | 0.96 | 0.81 |
| 47 | озеро песня шея | лебединый | 0.46 | 0.12 | 0.42 | 0.36 | 0.88 | 59 | 4.82 | 0.63 | 1 | 0.83 |
| 48 | горизонт жизнь фронт | линия | 0.52 | 0.1 | 0.38 | 0.37 | 0.83 | 60 | 6.97 | 0.58 | 0.97 | 0.75 |
| 49 | постный товар должностной | лицо | 0.28 | 0.4 | 0.32 | 0.67 | 0.73 | 60 | 16.44 | 0.41 | 0.53 | 0.49 |
| 50 | горе суп шелуха | луковый | 0.68 | 0.17 | 0.15 | 0.2 | 0.85 | 60 | 7.25 | 0.61 | 0.95 | 0.67 |
| 51 | картина машинный каша | масло | 0.83 | 0.07 | 0.1 | 0.33 | 0.87 | 60 | 7.76 | 0.6 | 0.94 | 0.71 |
| 52 | угол болезнь услуга | медвежий | 0.47 | 0.18 | 0.35 | 0.5 | 0.72 | 60 | 10.23 | 0.32 | 0.75 | 0.5 |
| 53 | сон море петля | мертвый | 0.38 | 0.26 | 0.36 | 0.55 | 1 | 42 | 7.4 | 0.69 | 1 | 0.76 |
| 54 | зимний медовый убывающий | месяц | 0.77 | 0.05 | 0.18 | 0.2 | 0.83 | 60 | 5.34 | 0.41 | 0.89 | 0.69 |
| 55 | колено капля открытый | море | 0.35 | 0.33 | 0.32 | 0.53 | 0.85 | 60 | 21.02 | 0.62 | 0.76 | 0.51 |
| 56 | задний древо собираться | мысль | 0.18 | 0.38 | 0.43 | 0.77 | 0.65 | 60 | 36.2 | 0.45 | 0.55 | 0.43 |
| 57 | правда протянуть делать | ноги | 0.31 | 0.39 | 0.31 | 0.66 | 0.75 | 59 | 11.09 | 0.61 | 0.78 | 0.62 |
| 58 | кошмар бабочка мгла | ночной | 0.89 | 0.08 | 0.03 | 0.18 | 0.84 | 38 | 08.09 | 0.59 | 0.91 | 0.75 |
| 59 | смена бабочка рубашка | ночной | 0.43 | 0.1 | 0.48 | 0.45 | 0.79 | 42 | 9.99 | 0.56 | 0.94 | 0.61 |
| 60 | дым игра меч | огонь | 0.25 | 0.45 | 0.3 | 0.75 | 0.53 | 60 | 13.51 | 0.4 | 0.47 | 0.27 |
| 61 | бритва боль блюдо | острый | 0.77 | 0.08 | 0.15 | 0.25 | 0.78 | 60 | 7.47 | 0.54 | 0.98 | 0.62 |
| 62 | мальчик дамский облизать | пальчик | 0.85 | 0.1 | 0.05 | 0.23 | 0.8 | 60 | 8.89 | 0.49 | 0.78 | 0.33 |
| 63 | дружеское рычаг гора | плечо | 0.52 | 0.27 | 0.22 | 0.6 | 0.67 | 60 | 12.16 | 0.48 | 0.65 | 0.33 |
| 64 | напольный шоколад электрический | плитка | 0.52 | 0.28 | 0.2 | 0.47 | 0.77 | 60 | 12.43 | 0.58 | 0.81 | 0.76 |
| 65 | катушка покой чаша | полный | 0.38 | 0.2 | 0.42 | 0.7 | 0.6 | 40 | 12.32 | 0.33 | 0.53 | 0.4 |
| 66 | невезение прибой препятствия | полоса | 0.32 | 0.27 | 0.41 | 0.54 | 0.76 | 59 | 11.09 | 0.79 | 0.84 | 0.62 |
| 67 | снимать перо высший | проба | 0.29 | 0.37 | 0.34 | 0.46 | 0.89 | 65 | 4.19 | 0.53 | 1 | 0.75 |
| 68 | карандаш число предложение | простой | 0.62 | 0.17 | 0.22 | 0.27 | 0.87 | 60 | 8.96 | 0.73 | 0.97 | 0.62 |
| 69 | эфир речь линия | прямая | 0.82 | 0.03 | 0.15 | 0.15 | 0.82 | 60 | 4.52 | 0.53 | 0.98 | 0.6 |
| 70 | жесткость вопрос бес | ребро | 0.42 | 0.37 | 0.22 | 0.68 | 0.65 | 60 | 17.26 | 0.76 | 0.8 | 0.31 |
| 71 | бык изобилие скрутить | рог | 0.91 | 0.04 | 0.04 | 0.26 | 0.78 | 46 | 4.9 | 0.57 | 0.93 | NA |
| 72 | умывать умелый лес | руки | 0.9 | 0.07 | 0.03 | 0.2 | 0.82 | 60 | 8.51 | 0.54 | 0.81 | 0.5 |
| 73 | пила мясо красный | рыба | 0.22 | 0.42 | 0.37 | 0.65 | 0.63 | 60 | 13.54 | 0.62 | 0.77 | 0.49 |
| 74 | новость зелень рана | свежий | 0.79 | 0.1 | 0.12 | 0.21 | 0.93 | 42 | 8.49 | 0.55 | 1 | 0.71 |
| 75 | перец жизнь вата | сладкий | 0.71 | 0.1 | 0.2 | 0.15 | 0.83 | 41 | 8.76 | 0.41 | 0.93 | 0.75 |
| 76 | куры грех нервный | смех | 0.68 | 0.22 | 0.1 | 0.4 | 0.72 | 60 | 7.11 | 0.51 | 0.83 | 0.39 |
| 77 | сено съесть зарыть | собака | 0.58 | 0.17 | 0.25 | 0.47 | 0.83 | 60 | 7.77 | 0.54 | 0.89 | 0.56 |
| 78 | любовь министры дружеский | совет | 0.27 | 0.3 | 0.43 | 0.58 | 0.8 | 60 | 22.79 | 0.56 | 0.81 | 0.63 |
| 79 | рана земля пуд | соль | 0.77 | 0.07 | 0.17 | 0.35 | 0.78 | 60 | 07.09 | 0.54 | 0.89 | 0.29 |
| 80 | книги водка складывать | стопка | 0.52 | 0.28 | 0.2 | 0.41 | 0.85 | 61 | 9.25 | 0.66 | 1 | 0.76 |
| 81 | костюм учитель ошейник | строгий | 0.34 | 0.3 | 0.36 | 0.61 | 0.67 | 61 | 10.97 | 0.43 | 0.81 | 0.41 |
| 82 | баловень улыбка уйти | судьба | 0.67 | 0.2 | 0.13 | 0.52 | 0.63 | 60 | 06.04 | 0.35 | 0.78 | 0.22 |
| 83 | небесный масса рубашка | тело | 0.3 | 0.38 | 0.32 | 0.67 | 0.68 | 60 | 25.12 | 0.72 | 0.83 | 0.44 |
| 84 | зрение жирный опора | точка | 0.43 | 0.43 | 0.13 | 0.57 | 0.82 | 60 | 15.13 | 0.54 | 0.88 | 0.75 |
| 85 | плод праведный сизиф | труд | 0.7 | 0.08 | 0.22 | 0.33 | 0.78 | 60 | 6.33 | 0.33 | 0.88 | 0.44 |
| 86 | острый_задний_палата | ум | 0.43 | 0.27 | 0.3 | 0.58 | 0.82 | 60 | 10.15 | 0.65 | 0.92 | 0.59 |
| 87 | дуть мотать сам | ус | 0.9 | 0.1 | 0 | 0.22 | 0.78 | 60 | 6.74 | 0.57 | 0.87 | 0.4 |
| 88 | чуткий вянуть лапша | уши | 0.83 | 0.13 | 0.03 | 0.3 | 0.83 | 60 | 10.5 | 0.58 | 0.86 | 0.89 |
| 89 | карточный объектив внимание | фокус | 0.4 | 0.38 | 0.22 | 0.62 | 0.82 | 60 | 12.27 | 0.83 | 0.96 | 0.62 |
| 90 | полный конь пустить | ход | 0.18 | 0.53 | 0.28 | 0.75 | 0.67 | 60 | 6.38 | 0.27 | 0.55 | 0.47 |
| 91 | борщ душ война | холодный | 0.66 | 0.09 | 0.25 | 0.3 | 0.93 | 44 | 10.4 | 0.59 | 1 | 0.87 |
| 92 | гонять крепкий Иван | чай | 0.52 | 0.1 | 0.38 | 0.33 | 0.92 | 60 | 5.95 | 0.71 | 0.97 | 0.86 |
| 93 | битый тихий потеха | час | 0.85 | 0.08 | 0.07 | 0.18 | 0.88 | 60 | 5.8 | 0.59 | 1 | 0.89 |
| 94 | юмор список работа | черный | 0.78 | 0.15 | 0.07 | 0.22 | 0.87 | 60 | 7.95 | 0.64 | 0.98 | 0.42 |
| 95 | бедность характер подводить | черта | 0.48 | 0.25 | 0.27 | 0.47 | 0.88 | 60 | 15.41 | 0.79 | 0.97 | 0.68 |
| 96 | воздушный земной магический | шар | 0.65 | 0.05 | 0.3 | 0.17 | 0.95 | 60 | 6.98 | 0.64 | 1 | 0.9 |
| 97 | часы банк сыр | швейцарский | 0.54 | 0.27 | 0.19 | 0.36 | 0.88 | 59 | 5.36 | 0.5 | 0.94 | 0.83 |
| 98 | дать сесть петля | шея | 0.22 | 0.41 | 0.37 | 0.64 | 0.75 | 59 | 13.54 | 0.62 | 0.85 | 0.59 |
| 99 | распускать вражеский пламя | язык | 0.18 | 0.27 | 0.55 | 0.63 | 0.7 | 60 | 12.19 | 0.64 | 0.91 | 0.46 |
| 100 | пиво долгий сыграть | ящик | 0.67 | 0.2 | 0.13 | 0.25 | 0.88 | 60 | 7.12 | 0.5 | 0.92 | 0.62 |

Supplementary Material should be uploaded separately on submission. Please include any supplementary data, figures and/or tables. All supplementary files are deposited to FigShare for permanent storage and receive a DOI.

Supplementary material is not typeset so please ensure that all information is clearly presented, the appropriate caption is included in the file and not in the manuscript, and that the style conforms to the rest of the article. To avoid discrepancies between the published article and the supplementary material, please do not add the title, author list, affiliations or correspondence in the supplementary files.

# Supplementary Material 1

*Basic principles for the problems’ selection*

For our version of the Russian RAT, the problems were selected according to two main criteria:

1. Each word of the triad connects with the solution word, but the connection is distant and, if possible, different for stimulus words. If this criterion is met, participants will presumably switch between different weakly related semantic areas while searching for connections.
2. There can be only one solution.

The version of the test that best meets these criteria is the CRAT (Bowden and Jung-Beeman, 2003) because the items in this test are composed of words that form either a compound word or an expression with the solution word. For instance: the problem, “age mile sand,” form the compounds, stone age, milestone, and sandstone with the solution word, stone. In addition, CRAT triads have only one correct answer with rare exceptions.

Because compound words are not typical of the Russian language, we preferred to use expressions to create problems (following the previous Russian version of the RAT; see Valueva and Belova, 2011; Valueva and Lapteva, 2021). It is worth noting that the expressions used to create problems are heterogeneous in their composition. Four types of expressions are distinguished: phraseological fusion (собаку съесть - means “he knows the subject inside out”), phraseological unity (буря в стакане воды - a storm in a teacup), phraseological combination (жар-птица - firebird), phraseological collocation (домашнее животное - pet) (according to Vinogradov’s typology [Vinogradov, 1977], which was subsequently supplemented by Shansky [1985]). The triads were composed so that each word in the triad would form an expression with the solution, and the stimulus words would be unrelated or weakly related to each other. In some of the triads, polysemantic words were used as the target word. In this case, the stimulus words referred to different meanings of the solution word.

The triad could include nouns, adjectives, and verbs. The solution word could be either a noun or an adjective. Because in the Russian language, the words agree with each other by changing their form, we decided to use the initial form of the stimulus words in all cases: verbs in an indefinite form, adjectives in the masculine, singular, nominative form, and nouns in the nominative, singular, or plural forms.

# Supplementary Material 2

*Instructions*

You will be shown triads of words. Your task is to choose such a word that can form a combination with each word from the triad (to form a fixed expression). For example, for a triad of words "HOOD," "CROSS," "SQUARE," the word-answer is "RED": (Red Hood, red cross, Red Square). You can also change the words grammatically: change the gender or use the necessary prepositions.

There will be 50 problems. The maximum time to solve each problem is 1 minute. If you find the answer earlier, press the SPACEBAR. Then you can enter your answer. After entering your answer, press the ENTER key.

You will be asked to answer a few questions after each problem. Use RIGHT and LEFT arrows to answer. The correct answer will be shown so you can check your responses.

Press SPACEBAR to start.

*An English translation is provided. Instructions were presented in Russian. See the original instructions at OSF:* [*https://osf.io/zumnr/*](https://osf.io/zumnr/)
